# Gene discovery for motile cilia disorders: mutation spectrum in primary ciliary dyskinesia and discovery of mutations in *CCDC151*

**DOI:** 10.1186/2046-2530-4-S1-P30

**Published:** 2015-07-13

**Authors:** A Onoufriadis, R Hjeij, CM Watson, CE Slagle, NT Klena, GW Dougherty, M Kurkowiak, NT Loges, CP Diggle, NF Morante, GC Gabriel, KL Lemke, Y Li, P Pennekamp, T Menchen, JK Marthin, D Mans, SJ Letteboer, C Werner, T Burgoyne, C Westermann, A Rutman, IM Carr, C O'Callaghan, E Moya, EMK Chung, UK10K, E Sheridan, KG Nielsen, R Roepman, RD Burdine, CW Lo, H Omran, H Mitchison

**Affiliations:** 1 Kings College London, London, UK; 2 University Children's Hospital Muenster, Muenster, Germany; 3 University of Leeds, Leeds, UK; 4 Princeton University, Princeton, NJ, USA; 5 University of Pittsburgh, Pittsburgh, PA, USA; 6 Copenhagen University Hospital, Copenhagen, Denmark; 7 Radboud University Medical Center, Nijmegen, The Netherlands; 8 UCL Institute of Ophthalmology, London, UK; 9 University of Leicester, Leicester, UK; 10 UCL Institute of Child Health, London, UK; 11 Bradford Royal Infirmary, Bradford, UK; 12 uk10k.org.uk, Cambridge, UK

## 

We present a stratification of the genetic basis of primary ciliary dyskinesia (PCD), based on screening >230 individuals for gene mutations using various approaches including whole exome sequencing. PCD is a genetically heterogeneous recessive ciliopathy, characterized by chronic lung disease and laterality and fertility defects arising from cilia and sperm dysmotility. Most PCD is caused by loss of the ciliary outer dynein arm motors (ODA) essential for motility, arising from mutations in ODA subunits or ODA docking and targeting proteins. Gene panel resequencing of candidate ciliopathy genes in affected children from a consanguineous Bedouin-Arabic family has recently revealed a homozygous protein truncating variant in *CCDC151 *(c.925G>T; p.Glu308*). Parallel exome sequencing combined with autozygosity mapping in a consanguineous UK-Pakistani-origin family highlighted a large autozygous region on chr 19p13 harbouring a homozygous *CCDC151 *protein-truncating variant (c.1256C>T; pSer419*). Sanger sequencing of *CCDC151 *in 150 more PCD cases identified another individual carrying c.925G>T. Transmission electron microscopy of respiratory cilia from individuals carrying *CCDC151 *mutations showed loss of ODA. Consistent with laterality defects in these individuals, we find *Ccdc151 *expressed in vertebrate left-right organizers. Both homozygous zebrafish and mouse *Ccdc151*-deficient mutants display situs defects associated with complex heart defects. Immunofluorescence analysis in patients shows that *CCDC151 *mutations abolish assembly of CCDC151 into respiratory cilia, and furthermore cause a failure in assembly of the ODA component DNAH5 and ODA docking proteins CCDC114 and ARMC4. We conclude that *CCDC151 *mutations appear to cause PCD by disruption of the axonemal ODA docking complex machinery.

